# Integrating Suspect and Nontargeted Screening for Food Safety Testing: A Case Study of Bee Pollen Supplements

**DOI:** 10.1021/acs.analchem.6c02768

**Published:** 2026-07-15

**Authors:** Susannah M. Heeren, Nynke I. Kramer, Laura Righetti

**Affiliations:** † Laboratory of Organic Chemistry, 4508 Wageningen University & Research, Wageningen 6708 EW, The Netherlands; ‡ Division of Toxicology, Wageningen University & Research, Wageningen 6708 EW, The Netherlands; § Wageningen Food Safety Research, Wageningen University and Research, Wageningen 6708 WB, The Netherlands

## Abstract

Natural food supplements, including bee pollen supplements, are increasingly popular, yet they may hide safety issues arising from concentrated chemicals, ranging from natural toxins to anthropogenic contaminants. To assess their safety, methods are needed to chemically characterize both their exposome and metabolome. Hence, we developed and validated a nontargeted screening method based on liquid chromatography high-resolution mass spectrometry (NTS LC-HRMS), combined with computational strategies to comprehensively annotate and simultaneously (semi)­quantify contaminants and bioactive compounds in bee pollen. The method was validated for 15 target compounds spiked at 0.3–3 mg/kg in the bee pollen, including pesticides, pyrrolizidine alkaloids, and mycotoxins (log *P* range of −1.4 to 5). These compounds were accurately annotated using the developed nontargeted workflow (100% accuracy when MS2 data were acquired) and semiquantified with a mean prediction error of 2.42. Concurrently, the method successfully annotated more than 4000 compounds in bee pollen by integrating suspect screening and nontargeted screening approaches. These results demonstrate the robustness and added value of integrated targeted and nontargeted screening strategies applicable to complex food matrices, effectively replacing several matrix-specific targeted methods. Moreover, this comprehensive characterization provides crucial information for the next steps of the risk assessment.

## Graphical abstract

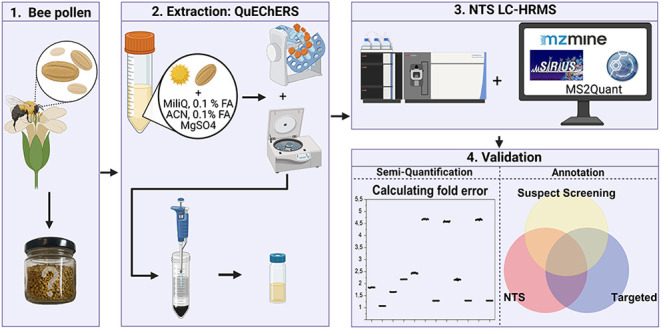

## Introduction

Access to safe food is considered so essential to human health that it is established as a human right.^1^ To secure the availability of safe food in Europe, the European Commission and the European Food Safety Authority have been working together to comply with the General Food Law Regulation^2^ by implementing thorough risk assessments.^3–5^ Curiously, these vast assessments are not as strongly legislated for food supplements, and thus, these supplements may pose a potential health risk.^6^ In recent years, numerous medical cases have been reported that highlight the association between adverse health effects and the consumption of various food supplements, such as specialty supplements (e.g., fish oils) and herbal/botanicals (e.g., bee pollen),^6,7^ implying that the health risks associated with natural food supplements should not be overlooked. Thankfully, the authorities have recently acknowledged these risks and added a selection of food supplements to the Commission Regulation (EU) 2023/915 in April 2023.^8^

One type of natural food supplement included in this regulation is pollen-based food supplements, also known as bee pollen supplements. Bee pollen supplements are growing in popularity due to both their nutritional (e.g., proteins, minerals, and fats) and medical (e.g., anti-inflammatory, antioxidant, and immune-enhancing) properties.^9–12^ However, only a limited number of studies have been conducted regarding potential adverse health effects.^12–18^ This is unfortunate given that numerous studies identified hazardous contaminants, such as mycotoxins,^12,14,19–21^ pesticides,^16,19,22,23^ and pyrrolizidine alkaloids,^12,24,25^ in bee pollen. Of these contaminants, only the pyrrolizidine alkaloids are included in the 2023 legislation.

Today’s gold standard for detecting such contaminants in food commodities is targeted liquid chromatography mass spectrometry (LC-MS). However, this approach comes with its limitations. One limitation is the necessity of analytical standards and prior knowledge on the composition of the sample. These analytical standards are not always commercially available, or are simply too expensive. A second limitation is the tunnel vision of a targeted approach. While the focus is on a selected set of compounds, many other compounds present are neglected. This is especially a bottleneck when working with complex mixtures, such as bee pollen. For example, it is known that the interaction between certain mycotoxins and bioactive compounds can result in synergistic effects.^26–28^ Ignoring such interactions will inevitably result in an inaccurate risk assessment. To prevent such inaccuracy, new methods are needed that are capable of the simultaneous detection of many compounds of different chemical classes. One approach is the use of nontargeted screening liquid chromatography high-resolution mass spectrometry (NTS LC-HRMS) combined with computational strategies to annotate and (semi)­quantify the detected chemicals.^29,30^

Currently, NTS LC-HRMS methods are mainly used within environmental research,^31,32^ and thus applied to less complex matrices, such as water. Only recently researchers have started to apply suspect screening (SS) LC-HRMS for different food commodities and supplements,^33,34^ which represent substantially more complex matrices.

However, these studies focus on only one class of chemicals. In our study, we broaden the scope of NTS by combining it with SS and targeted analysis to explore the chemical exposome and metabolome of bee pollen supplements. The ability to annotate many unknown knowns and predict their concentrations within a single run makes this approach a compelling proof of concept in the world of food toxicology and next-generation risk assessments.^35–37^ However, NTS LC-HRMS methods do have their own challenges. One such challenge is the large number of complex data generated, from which relevant and reliable information needs to be extracted. To achieve this, we developed and validated a novel NTS workflow, which includes the sample extraction, NTS LC-HRMS method, and data processing including semiquantification. A validated NTS LC-HRMS method provides a way to comprehensively characterize food supplements and secure reliable and semiquantitative data for risk assessments to food toxicologists.

## Experimental Section

### Chemicals and Reagents

All reagents and solvents used in this work were of HPLC grade unless otherwise stated. Ultrapure water was obtained from a Milli-Q IQ 7000 system. Methanol (MeOH), acetonitrile (ACN), and ethanol (EtOH) were purchased from Macron, Sigma-Aldrich, and Boom, respectively. The 99% formic acid (FA) (LC-MS grade) and ammonium acetate (LC-MS ultra) were purchased from Fisher Scientific B.V. Ammonium formate (LC-MS grade) was purchased from Sigma-Aldrich. Two stock solutions (stock 1 (*N* = 20) and stock 2 (*N* = 10)) with different bioactive compounds and contaminants were prepared for the development, optimization, and validation of the NTS LC-HRMS method. The composition of each stock solution is available in SI-1.

### Bee Pollen Samples

Commercially available bee pollen samples were collected from retailers from different geographical locations. After collection, the samples were milled until they were homogeneous and stored at 4 °C until further use. A set of 10 bee pollen samples was mixed in a ratio of 1:10 weight% to obtain a representative sample. Moreover, 11 bee pollen samples were measured with the developed NTS method and an in-house SS method^38^ as part of the validation. The targeted analysis of pesticides and pyrrolizidine alkaloids included an additional 9 and 6 bee pollen samples, respectively.

The pooled bee pollen were used to prepare three samples dedicated to the development, optimization, and validation of the extraction, detection and processing method: (1) QC1, nonspiked bee pollen; (2) QC3, bee pollen spiked with calibrants (15 contaminants and toxins, stock 1) with a concentration of 100 μg/L for each compound; and (3) VAL, bee pollen spiked with an additional set of standards (10 contaminants and toxins, stock 2). The end concentration of the contaminants present in the VAL samples was 33 μg/L for all (see SI-1), except acetamiprid, phosmet, and aflatoxin M1 (333, 342, and 5 μg/L, respectively).

### Sample Extraction

Before extraction, the bee pollen samples were spiked with L-tryptophan-d5 to a final concentration in a vial of 500 μg/L (Cayman Chemical). After extraction, the extracts were spiked with U-[13C34]-fumonisin B1 and U-[13C18] Zearalenone (BioPure) with a final concentration of 200 μg/L and 100 μg/L, respectively.

Two extraction methods were explored to find the most suitable method: solid–liquid extraction based on solvent extraction and modified QuEChERS.

#### Solvent Extraction

Five different solvents were tested: 75% ACN in water, 75% EtOH in water, 75% MeOH in water, EtOH:ACN (45:30 v/v) in water, and MeOH:ACN (45:30 v/v) in water. All solvents contained 0.1% FA to enhance the extraction efficiency. Half a gram of bee pollen was weighted to which 5 mL of one of the above-mentioned solvents was added. Next, the samples were shaken for 45 min (shaking plate, 300 rpm), followed by ultrasonication for 15 min. The extract was transferred to a 1.5 mL Eppendorf tube and centrifuged with a tabletop centrifuge. The supernatant was evaporated to dryness under a stream of nitrogen and subsequently stored at −20 °C until analysis. The dried extract was reconstituted in 50% ACN prior to LC-HRMS analysis.

#### Modified QuEChERS

Bee pollen samples (0.5 g) were prewetted with 4 mL Milli-Q water (containing 0.1% FA). After 30 min, 4 mL of cold ACN (containing 0.1% FA) was added, followed by thorough shaking by hand and 1 min on the overhead shaker (300 rpm). Next, 2.4 g MgSO_4_ (VWR chemicals) was added, followed by another minute on the overhead shaker. The samples were placed in a refrigerator (4 °C) to cool for 5 min, followed by centrifugation (4 min, 4000 rpm, 10 °C). The supernatant was transferred to a 15 mL falcon tube and dried under a stream of nitrogen and stored at −20 °C until further use. On the day of analysis, the samples were reconstituted in 80% MeOH.

### Instrumental Analysis

In this work, targeted analysis, NTS and SS were exploited. The details for the targeted analysis are available in SI-2, while the SS method was as described by Padilla-González et.al.^38^

For the NTS LC-HRMS method, a Thermo Scientific Orbitrap IQ-X Tribrid Mass Spectrometer coupled to a Thermo Scientific LC system was used. Chromatographic separation was obtained with an Acquity UPLC HSS T3 1.8 μm (2.1 mm × 100 mm) column and an Acquity UPLC HSS T3 1.8 μm VanGuard Pre-Column (2.1 mm × 5 mm), both from Waters. The mobile phase consisted of 5 mM ammonium acetate in Milli-Q, 0.1% FA (Solvent A) and 5 mM ammonium acetate in MeOH, 0.1% FA (solvent B). The total run time was 17 min, starting at 1% B. After 1.5 min of isocratic hold, the organic phase increased to 100% B over the course of 9.5 min. After a hold of 3 min at 100% B, the system returned to the starting conditions of 1% B in 0.3 min. The re-equilibration time was 2.7 min. The flow rate was set to 0.3 mL/min and the column oven to 40 °C.

Heated electrospray ionization (HESI) was utilized in both positive and negative ionization mode with the following settings: sheath gas: 50 Arb; auxiliary gas: 10 Arb; sweep gas: 1 Arb; static spray voltage: 3500 V (positive ion mode) and 2500 V (negative ion mode); ion transfer tube temperature: 325 °C; internal mass calibration: RunStartEasyIC. The scan range for both the full scan and MS2 measurements was set to *m*/*z* 100–1000 with an orbitrap resolution of 120 and 30 K, respectively. Dynamic exclusion was activated during the full scan for 6 s with a mass tolerance of 5 ppm. The AGC target was set to 40,000 a.u. and for the DDA to 5000 a.u. The RF lens was 60%. During the DDA measurements, the assisted collision energy mode was utilized with CE of 15, 30, and 45 eV with HCD.

### Evaluation Extraction and NTS LC-HRMS Method Performance

Both the extraction method and the NTS LC-HRMS method were validated. The performance of the extraction method was evaluated in terms of extraction recovery and matrix effects. For this purpose, the pooled bee pollen samples were spiked before and after extraction with Stock 1 (final concentration 150 μg/L). The LOD values of these compounds were evaluated by spiking bee pollen extracts with 50–0.005 μg/L, with factor 10 steps.

The NTS LC-HRMS method was developed on the basis of 24 standards. These 24 standards were selected based on their log *P* and their presence in bee pollen according to the literature (Table S3). Moreover, it was desired that the selected standard represented the chemical space of the bee pollen (Figure 4). Different mobile phases were tested to obtain optimal elution and ionization of these 24 compounds. The response factor (eq 1) was calculated for each standard to establish the most suitable mobile phase.

1 $$response\;factor\;\left(RF\right)=\frac{peak\;ar\mathrm{ea}\;s\tan dard}{concentration\;s\tan dard}$$

The sensitivity, reproducibility, and retention time shifts of the NTS LC-HRMS method were evaluated by analyzing QC3 and QC1, every 12 h over the course of 4 days (*N* = 8).

### Data Processing

The annotation and semiquantification of the proposed workflow (Figure 1) were evaluated by different approaches. The accuracy of the annotation was evaluated by (1) comparing the annotation via both open-source and vendor software, (2) annotation of spiked standards, (3) targeted analysis, (4) SS analysis, using the WFSR Food Safety Mass Spectral library (FSMS library),^38^ and (5) manual checking. The accuracy of the predicted concentration via semiquantification was evaluated by (1) calculating fold error of the contaminants used in the calibration model and identified with the targeted analysis, and (2) setting a concentration range for spiked contaminants not included in the calibration model – the validators (VAL).

**Figure 1:**
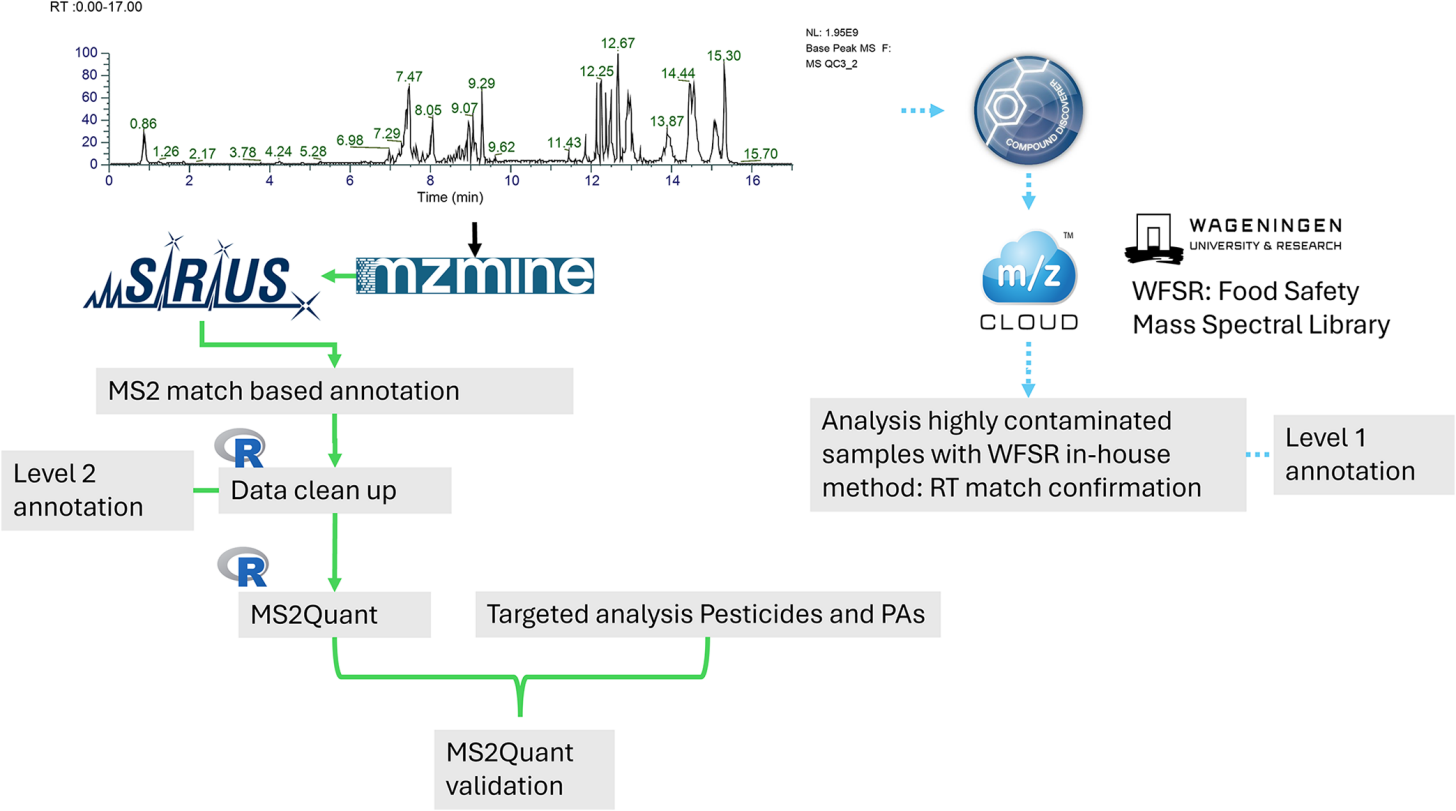
Developed and validated data processing workflow. Complementary approaches including both open source (SIRIUS) and vendor software (Compound Discoverer) were used to annotate unknown known compounds in bee pollen supplements. To obtain a level 1 annotation, suspect screening with the WFSR Food Safety Mass Spectral Library LC-HRMS method was implemented. Furthermore, the annotated compounds were semiquantified using the computational model MS2Quant. To evaluate and validate the predicted concentrations, targeted analysis of pesticides and pyrrolizidine alkaloids (PA) was executed.

#### Preprocessing

The bee pollen spiked with contaminants (QC3 and VAL) was first manually evaluated in Freestyle 1.8 SP1 (Thermo Scientific) to determine whether these compounds were detected, if an MS2 spectrum was acquired, and to confirm the match of the retention times with the standards. The raw data were subsequently converted to .MZML by ProteoWizard, with peak picking for both MS1 and MS2, followed by peak alignment with MZmine (version 4.3.0). The settings for MZmine are available in SI-3.

#### Annotation

Two pathways were explored for the annotation of the unknown knowns: (1) SIRIUS (version 5.8.6)^39–45^ and (2) compound discoverer (CD). The details for each software are available in Tables S1 and S2. After processing with SIRIUS, the data were screened in Rstudio (version 2024.04.2.764) to remove any features without a confidence score, a mass error of >5 ppm, and a CSI:FingerID Score of < −150. The R script is available in SI-4.

#### Semiquantification

MS2Quant (ref = changing models)^46^ was used in Rstudio (version 2024.04.2.764) to predict the concentrations of the features annotated by SIRIUS based on the ionization efficiency. MS2Quant uses calibration curves of compounds that were run together with the samples. In this study, 13 calibrants were included for the calibration model in positive ionization mode and 7 for negative ionization mode. All calibrants were in solvent and ranged between 0.25 and 2000 μg/L (Table S3). The following settings were usesd in MS2quant: organic modifier = MeOH; pH_aq = 4.8, and NH4 = 1. The accuracy of the concentration prediction was evaluated by calculating the fold error (eq 2, in which Conc. P refers to the predicted concentration and Conc. R to the quantified concentration) for the 13 contaminants. The fold error was additionally used to set a concentration range for the validators to assess its suitability.

$$\begin{array}{c} \mathrm{fold}\;\mathrm{error}=\frac{\mathrm{conc}.P}{\mathrm{conc}.R},\mathrm{if}\;\mathrm{conc}.P>\mathrm{conc}.R\\ =\frac{\mathrm{conc}.R}{\mathrm{conc}.P},\mathrm{otherwise} \end{array}$$ 2

## Results and Discussion

### Extraction Optimization

To ensure the most efficient extraction of the bee pollen, 5 different solvent extractions and modified QuEChERS were tested. The term modified QuEChERS is used as the d-SPE step is left out to avoid losing any other metabolites. The different extraction methods were compared based on the number of aligned, tentatively annotated, and semiquantified features (Figure S1A,B). For the two most favorable extraction methods (modified QuEChERS and ACN:MeOH) an additional set of 7 extractions was performed to determine which of these two is the most suitable. The results (Figure S1C,D) indicated that more features are consistently (CV% modified QuEChERS = 6% (ESI+) and 5% (ESI-); *N* = 7) extracted with the modified QuEChERS method than with ACN:MeOH (≈200 features more with modified QuEChERS). Additionally, the extracted chemical classes were evaluated for both approaches, to ensure the extraction of a broad range of chemical classes, including contaminants and bioactives (Figure 2). Figure 2 illustrates the outperformance of the modified QuEChERS for most chemical classes. Exceptions to this include chromanes, the chemical class of aflatoxins. This was expected as literature already indicated that a solvent and/or solid-phase extraction is the most suitable for aflatoxins.^14^ However, in this work, it was demonstrated that the aflatoxins are also extracted with good recovery (>80%) via modified QuEChERS (Figure S2).

**Figure 2:**
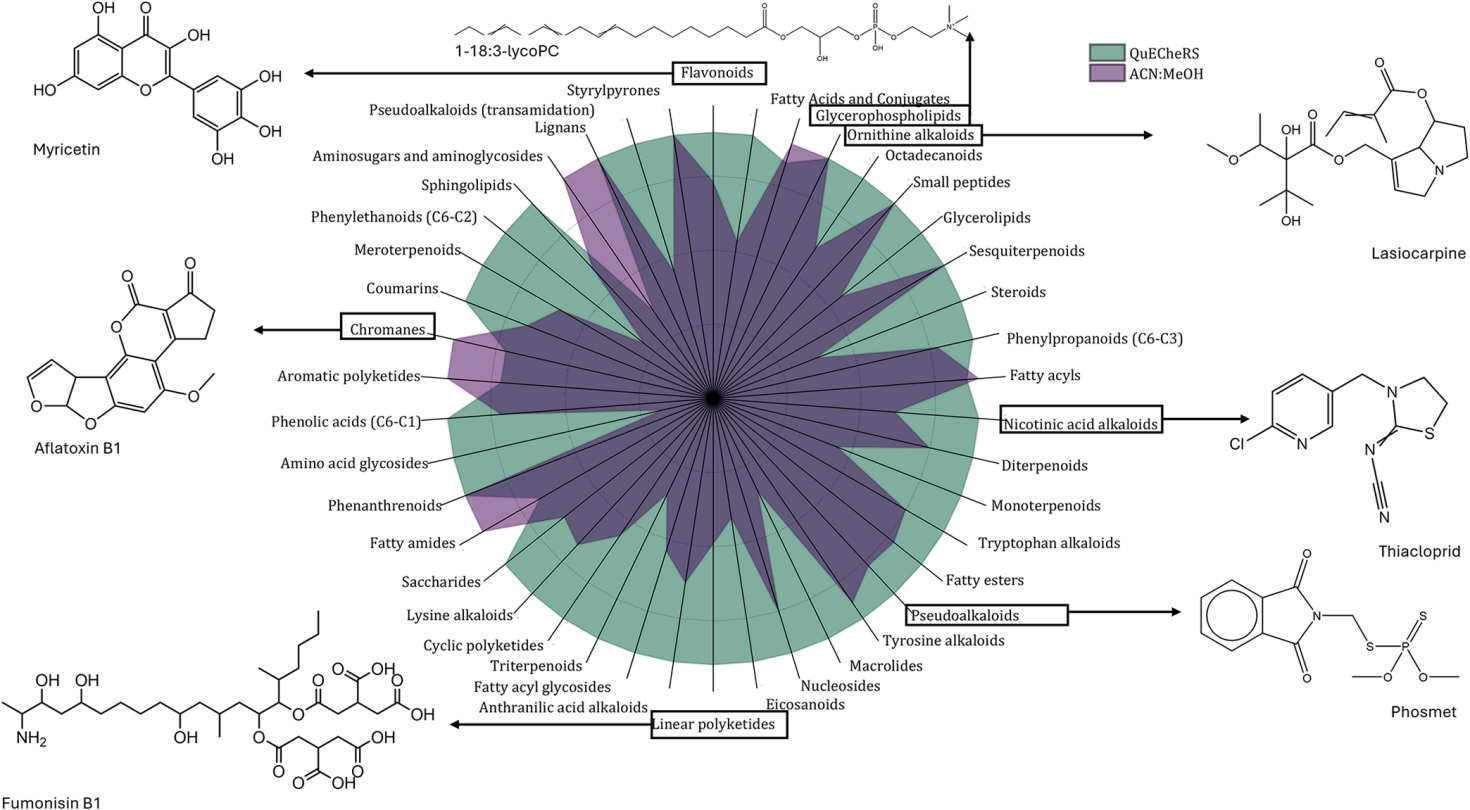
Spider graph comparing chemical classes extracted by modified QuEChERS (green) and ACN:MeOH (purple). The structures represent examples of contaminants and bioactive compounds present in the bee pollen.

### NTS LC-HRMS Method Optimization

The response of 24 compounds (Table S3) was tested using two different modifiers in the mobile phase, ammonium formate and acetate (Figure S3A). The results show that most compounds ionized efficiently under both buffering conditions (Figure S3A). Deoxynivalenol (DON) is the only compound that solely ionizes with ammonium acetate, which was therefore selected as the buffer. Moreover, different solvents were assessed: (1) ACN, 0.1% FA; (2) MeOH, 0.1% FA; and (3) ACN:MeOH (1:1 v/v), 0.1% FA. On the basis of the elution strength and polarity of the two organic modifiers, it was expected that the mixture of ACN and MeOH (Figure S3B, blue triangle) would be the most suitable. However, the response factors revealed that MeOH, 0.1% FA leads to a higher response factor (Figure S3B, red circle). These two arguments led to the decision to continue with MeOH and 0.1% FA as organic modifier for solvent B.

#### Extraction Recovery and Matrix Effects

The extraction recovery (ER) and matrix effects (ME) were evaluated for the 15 contaminants included in the development of the NTS LC-HRMS method (Figures S2A and S2B, respectively). The ER for this broad range of compounds (log *P* −1.4–5) ranged between 56% and 94%, confirming the suitability of the extraction method. However, most compounds encounter ion suppression (Figure S2B). An exception to this is PFOA, which suffers from a major ion enhancement. The reason behind this is unclear, but the ME of PFAS is known to vary quite a bit depending on the type of matrix.^47–49^ These results are therefore not surprising, especially with the very limited cleanup of the samples. To reduce the ME, a cleanup step (e.g., SPE) could be added during extraction. However, in this work this was not done due to the risk of removing compounds of interest. An alternative to a cleanup is to correct for the matrix effect after analysis, which is one of the challenges for NTS LC-HRMS methods. Recently, several groups have explored different approaches to overcome this challenge, such as the use of isotopically labeled standards and an individual sample-matched internal standard strategy.^50,51^

### Validation of Feature Annotation

To ensure the reliability of the feature annotation, spiked bee pollen was processed with both SIRIUS and CD (FSMS library and mzCloud). Moreover, the WFSR FSMS library SS LC-HRMS method was implemented to obtain a level 1 annotation for these known food contaminants.

#### Annotation Non Target Screening LC-HRMS Data

The annotation was evaluated using the contaminants (*N* = 15) spiked in QC3. Figure 3A demonstrates the number of features annotated with a level 1 or level 2 annotation identification confidence^52^ using SIRIUS and compound discoverer (FSMS library and mzCloud). It can be observed in Figure 3A (bold compounds) that 13 out of 15 spiked contaminants were accurately annotated by SIRIUS. Carbaryl and chlorpyrifos were not annotated by SIRIUS, as no MS2 was acquired because of low ion intensity in the MS1. This is one of the main limitations of using DDA-MS2, as without the fragmentation, there is no identification. To overcome this limitation, an ion trap was explored for acquiring more MS2 data. However, the low resolution of the thus resulting MS2 data made it unfit for SIRIUS. Additionally, when processing the data with CD, fewer features were annotated at a level 2 annotation (Figure S4). These two arguments together led to the decision to use the Orbitrap to acquire MS2 data.

**Figure 3:**
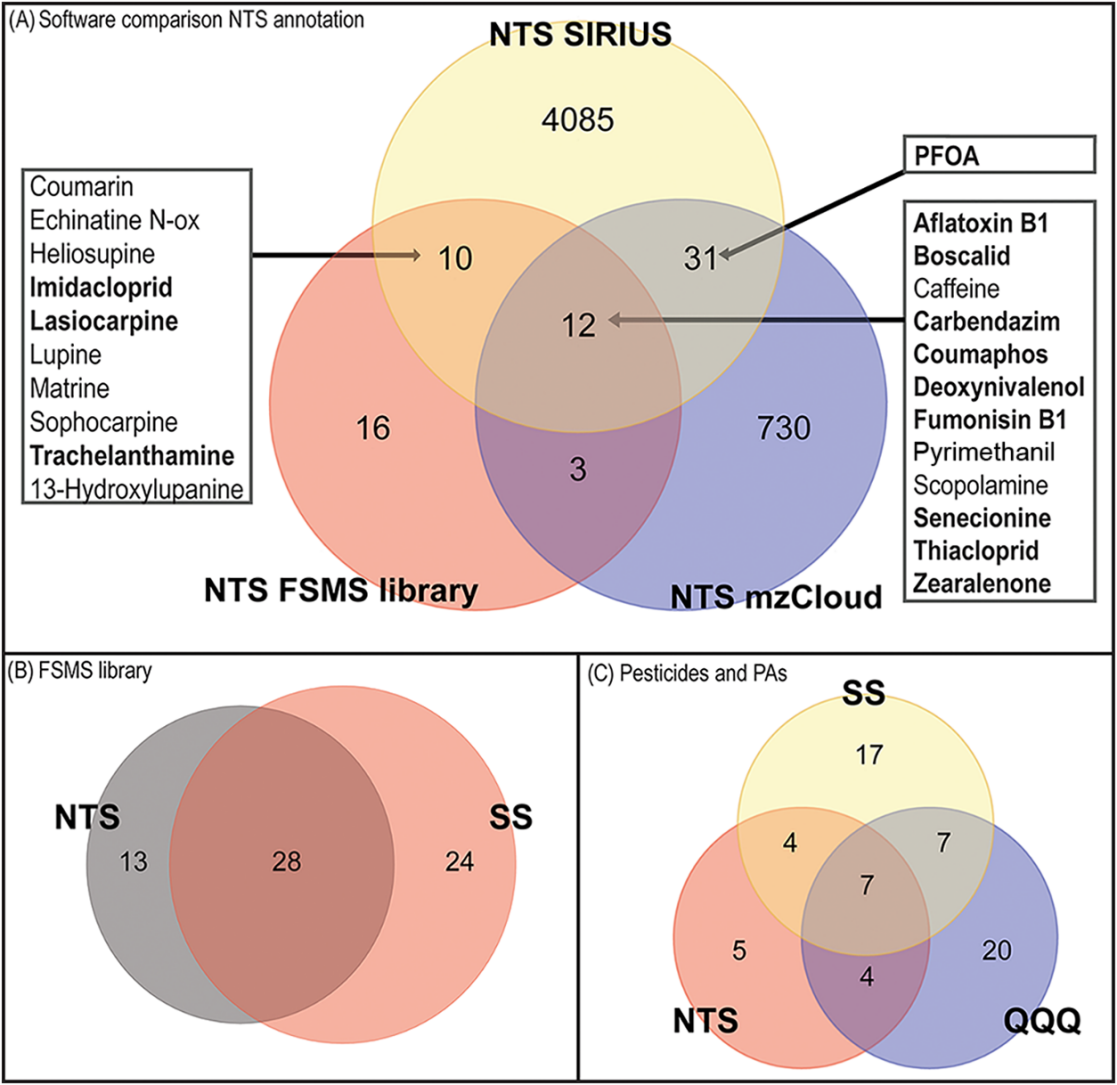
Venn diagrams for annotation comparison. (A) NTS feature annotation of 11 bee pollen samples and QC3 using SIRIUS and compound discoverer (FSMS library and mzCloud). (B) NTS and SS annotation comparison of known food contaminants identified with the FSMS library. (C) Comparison of pesticides and PAs identified via NTS, SS, and targeted analysis (QQQ), revealing that only 7 contaminants were identified by all three approaches. All Venn diagrams were generated in ORIGIN 2020b.

While processing the Orbitrap data with CD, not all compounds were annotated using the SS approach with the FSMS library and mzCloud. PFOA was not annotated with the FSMS library as the negative ionization mode is not included in the library. Lasiocarpine and trachelanthamine were identified with the FSMS library but not with the mzCloud. Further evaluation revealed that these two compounds are not present in the mzCloud, explaining their absence in the annotation. Imidacloprid is available in the mzCloud. However, as it was only a partial match, it was excluded as a positive annotation. Additionally, it can be observed in Figure 3A that there are an additional 16 known food contaminants identified only with the FSMS library. This finding emphasizes the need to use complementary annotation approaches to cover as much of chemical space as possible. However, it should be acknowledged that using multiple complementary annotations is time-consuming and generates a lot of data. While this is needed for some purposes, it might not be necessary for all of them. This necessity is further highlighted by visualization of the bee pollen chemical space (Figure 4). The UMAP graph represents the annotated compounds from 11 different bee pollen samples via SIRIUS (Figure 4, orange dots), mzCloud (Figure 4, pink dots), and the FSMS library (Figure 4, blue dots). The compounds found in the literature (Figure 4, green dots) and the standards used (Figure 4, black dots) are visualized in the UMAP together with the bee pollen samples, emphasizing that our developed method covers and has extended the chemical space of bee pollen. Additionally, it proves that the standards chosen to develop the NTS method are representatives of this chemical space.

**Figure 4:**
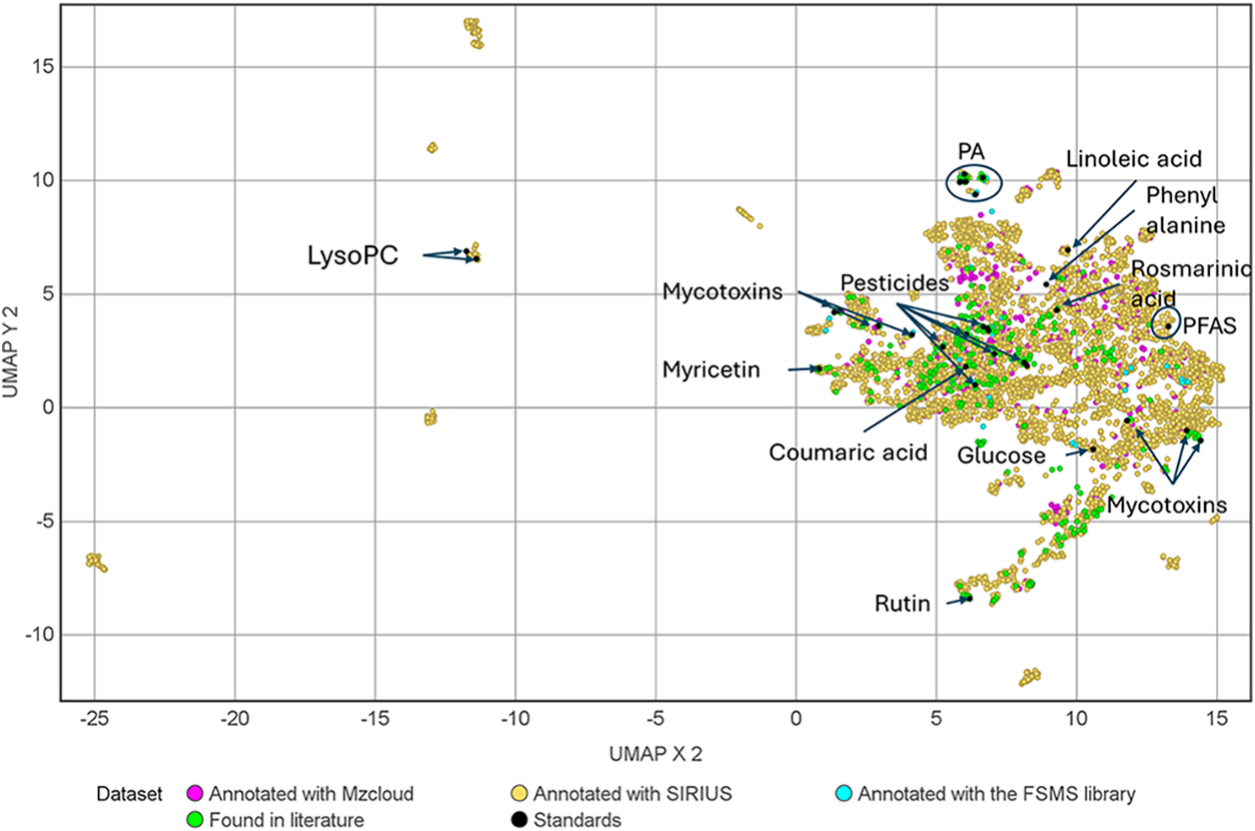
Chemical space visualization of the bee pollen metabolome and exposome. The black dots represent the standards used for the development and validation of the NTS LC-HRMS method. The green dots represent the bioactive compounds and contaminants previously reported in the literature. The remaining dots represent the compounds annotated with our workflow for 11 bee pollen samples (orange for SIRIUS, pink for mzCloud, and blue for the FSMS library). The small clusters annotated with SIRIUS (UMAP X2 −10 to −25 range) are all phospholipids. The UMAP plot was built using DataWarrior and is available on Zenodo (DOI: 10.5281/zenodo.19334300).

#### Annotation Suspect Screening LC-HRMS Data

Annotation via the FSMS library for the NTS LC-HRMS analysis was confirmed through the WFSR in-house developed SS LC-HRMS method.^38^ The data acquired were processed with the FSMS library, after which the annotations were compared with the NTS annotations (Figure 3B) (raw data available on Zenodo. DOI:10.5281/zenodo.19334300). The results confirmed the presence of 28 food contaminants. In addition, 24 other food contaminants were identified using the SS method. Further exploration of the NTS data revealed that of these 24 compounds, 11 compounds acquired matching MS2 with the NTS method. Yet, they were not identified with the FSMS library. It is unclear why this happened, especially since four of them were correctly annotated by SIRIUS (Table S4). Overall, these results indicate that the NTS method has appropriate sensitivity but also acknowledges the occurrence of false negative annotations. Concurrently, the NTS method revealed 13 food contaminants that were not identified with the SS method, supporting the sensitivity of the NTS method and emphasizing the importance of implementing complementary methods.

#### Targeted Analysis

To further evaluate the NTS annotation accuracy, targeted analysis of 300 pesticides and 78 pyrrolizidine alkaloids was done on a selection of bee pollen samples (*N* = 20 for pesticides and *N* = 17 for PAs). First, the list of identified pesticides and PAs was compared to those detected with the NTS and SS methods (Figure 3C). This exposed that from the identified pesticides and PAs, 9 PAs and 17 pesticides were not annotated by SIRIUS. Moreover, the comparison with the targeted analysis indicated that matrine and fenpropidin were false-positive in the SIRIUS annotation. This was later confirmed through the analysis of the standards with the NTS method (Figures S5–S7). The chromatograms show that the two standards have different retention times than the compounds present in the sample. This separation suggests that the compounds are isomers of fenpropidin and matrine. From a technical point of view, it makes sense that SIRIUS cannot distinguish between isomers solely on the basis of similar MS2. Moreover, these two cases highlight that this generic LC method can separate isomers, which is crucial when dealing with complex mixtures. Additionally, the majority of the 17 pesticides that were not annotated were detected with the NTS method. However, no MS2 was obtained. In contrast to the missing compounds, the results revealed the annotation of pesticides and PAs via the NTS and SS methods that were excluded from the targeted analysis. This emphasizes the power of annotating unknown knowns with both NTS and SS LC-HRMS.

### Validation of Semiquantification via MS2Quant

One of the major drawbacks in nontargeted analysis is the absence of quantified data, while this is typically crucial information in risk assessments. Therefore, in this work, we aimed to overcome this challenge by applying a semiquantification prediction model (MS2Quant) that uses the peak intensities to obtain semiquantified values for contaminants and bioactive compounds. These values can then be used to perform risk assessments not only for single compounds but also for complex mixtures. MS2Quant was used to semiquantify the features annotated via SIRIUS. As MS2Quant is a prediction model, two approaches were implemented to evaluate the accuracy and precision of the model: (1) evaluation of the prediction error with standards and (2) comparison of the semiquantified concentrations with quantified concentrations of bee pollen samples (targeted analysis).

It is worth noting that only the contaminants were used in this validation process. This was done since bee pollen naturally contains many bioactive compounds, making it difficult to obtain a blank matrix suitable for the validation.

#### Evaluation of Prediction Model with Standards

Pooled bee pollen was spiked with the calibrants and validators. The calibrants were used to calculate the fold error (eq 2) using the concentrations obtained with the calibration curve and MS2Quant. An average fold error of 2.5 was obtained with a maximum of 4.7 (Figure 5A). The performance of the model for DON could not be assessed, since no MS2 spectra were acquired. As the fold error for each calibrant lies below 5, it was expected that the trend would continue for the unknown knowns. To evaluate this hypothesis, we used the validators. For each compound, the expected and predicted concentrations were known. After comparison of the two values, it was clear that all compounds, except for spartioidine N-oxide, were underpredicted (Figure S8A). It is hypothesized that the overprediction of spartioidine N-oxide is due to potential coelution with other isomeric PAs (*e.g*., seneciphylline N-oxide).

**Figure 5:**
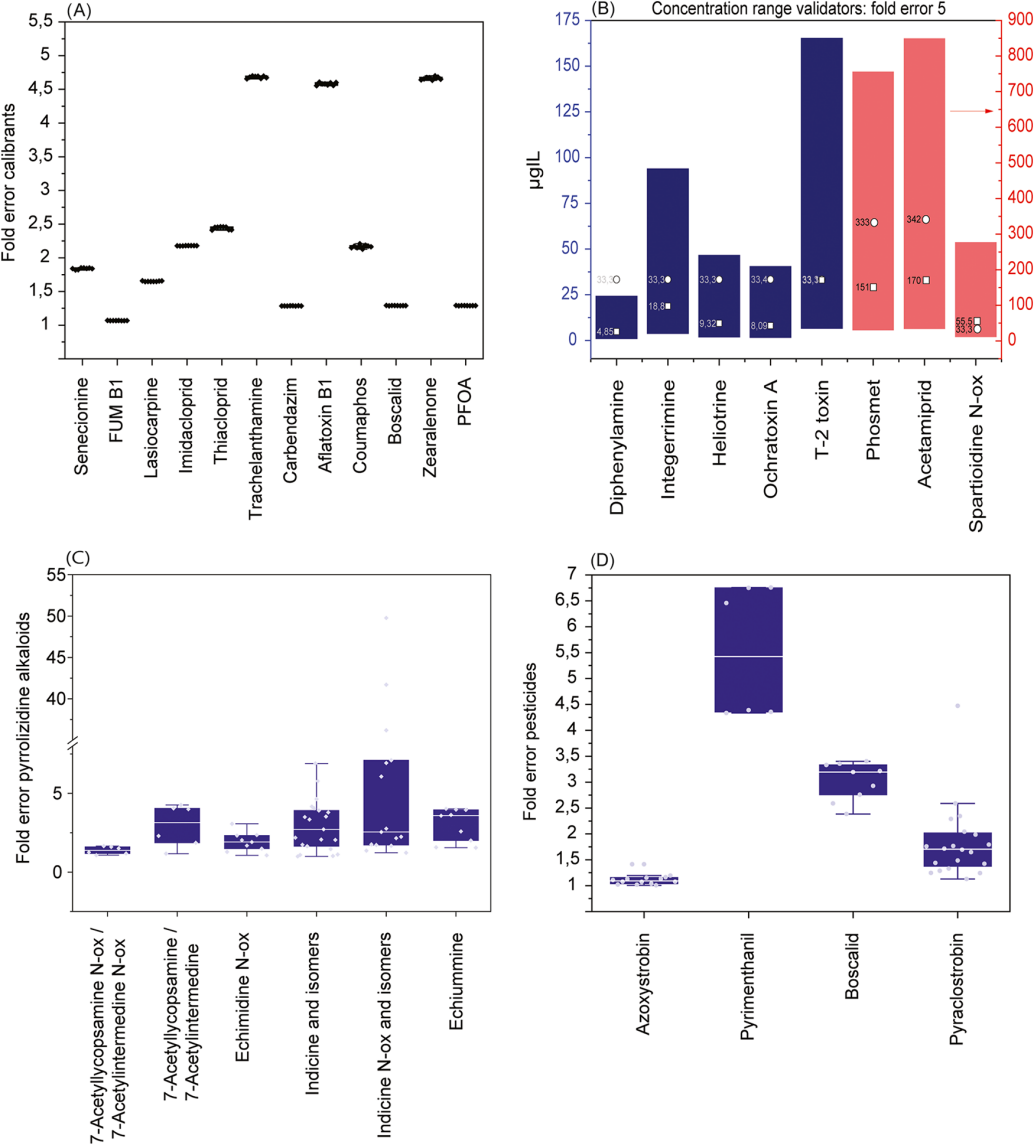
Evaluation of semiquantification via MS2Quant using the fold error: (A) Calculated fold errors of calibrants (*N* = 8). (B) set concentration range for validators based on the maximum observed fold error of 5. (C) Fold error derived from targeted analysis data of pyrrolizidine alkaloids. (D) Fold error derived from targeted analysis data of pesticides.

The broadly observed underprediction could be related to the observation of ion suppression in the calibrants (Figure S2A) or to a low extraction recovery. These factors were not considered for the validators. To take account of the under- and overprediction of the semiquantification, a concentration range was set for the compounds in which the true concentration is expected to be in. This range was set by determining the upper- and lower-bound by dividing or multiplying the predicted concentration with the fold error of 5 (Figure 5B) and 2.5 (Figure S8B). Figure 5B suggests that using the fold error of 5 is sufficient for most compounds. An exception is diphenylamine, for which the expected value is still above the upper bound. This implies that even with a restrained prediction error, the true concentration may not lie within the range. This is especially a risk when lower concentrations are used. To further investigate this, we evaluated the model on bee pollen samples.

#### Evaluation of Prediction Model with Bee Pollen Samples

The fold error was calculated for the pesticides and pyrrolizidine alkaloids detected with both targeted and nontargeted analysis (Figure 5C,5D). The fold error was above 5 for three out of ten contaminants, indicating that a fold error of 5 is not protective enough to set a concentration range. This is also confirmed by diphenylamine (Figure 5B). Based on these results, it was concluded that a fold error of 7 is more appropriate to use to set the concentration range for unknown knows. However, it should be stressed that the fold error gives a range in which the true concentration is expected to be in. For example, ochratoxin A was spiked at 33 μg/L, while the semiquantification resulted in 8 μg/L (Figure 5B). Implementing the fold error of 7 results in a range of 56–1.14 μg/L, which includes the true concentration of 33 μg/L. Even though the semiquantitative data cannot be implemented for further steps of risk assessment directly, it helps with prioritization of compounds of concern. To conclude, we would like to highlight that while this approach is not yet ready for a full risk assessment approach, it is the first step toward quantitative analysis. Without it, there are only peaks and no indication regarding concentration at all.

## Conclusions

In this study, we successfully developed a NTS method based on LC-HRMS, combined with a data analysis strategy to comprehensively annotate and simultaneously (semi)­quantify contaminants and bioactive compounds in bee pollen. An extensive validation step, often lacking in untargeted methodologies, was performed. This validation covered the entire analytical workflow from sample preparation and detection to compound annotation and (semi)­quantification. The proposed approach successfully captured the chemical space of bee pollen and enabled the annotation of over 4000 compounds across a wide polarity range. Additionally, we obtained semiquantitative data, including trace-level concentrations (6.10 × 10^–4^ to 3.03 × 10^–1^ μM), suitable for risk assessment purposes. Furthermore, the workflow is transferable to other food supplements. However, the extraction method should be optimized for the matrix of interest. Nevertheless, this transferability is paving the way for more holistic risk assessment strategies that account for the complex mixture of both contaminants and bioactive compounds present in food matrices, an aspect that has been largely overlooked until now.

## Supplementary Material



## Data Availability

The raw data for the SS and NTS LC-HRMS analysis is available on Zenodo (DOI:10.5281/zenodo.19334300).

## Notes

Stock solutions (SI-1); targeted analysis settings (SI-2); MZmine settings (SI-3); R script (SI-4); SIRIUS settings (Table S1); compound discoverer settings (Table S2); additional information on calibrants including the RT and linear range (Table S3); false negatives NTS method – annotation with FSMS library (Table S4); aligned, annotated and semiquantified features for different extraction methods (Figure S1); extraction recovery (A) and matrix effect (B) for the calibrants (Figure S2); evaluation of the different buffers (A) and organic solvents (B) tested during the development of the NTS HPLC-HRMS method based on the response factors in logarithmic scale (Figure S3); comparison of the ion trap and orbitrap to acquire MS2 (Figure S4); matrine MS1 (Figure S5); matrine MS2 (Figure S6); fenpropidin MS1 (Figure S7); (A) comparison between predicted and expected concentrations of Validators. (B) Set concentration range when using the average fold error of 2.5 (Figure S8) (PDF)
