# Supplementary material for: Integrating Suspect and Nontargeted Screening for Food Safety Testing: A Case Study of Bee Pollen Supplements
Source: Anal Chem. 2026 Jul 15;98(29):21732–41. doi: 10.1021/acs.analchem.6c02768 (PMC13425548; doi:10.1021/acs.analchem.6c02768)
Supplement: Supplementary file 1 [file ac6c02768_si_001.pdf]

Supplementary data - Integrating suspect and non-targeted screening for food safety testing: a case study of bee pollen supplements

Susannah M. Heeren [1,2,3], Nynke Kramer [2], Laura Righetti [1,3]*

[1]Laboratory of Organic Chemistry, Wageningen University & Research, Wageningen 6708 EW, The Netherlands.

[2]Division of Toxicology, Wageningen University & Research, Wageningen 6708 EW, The Netherlands.

[3]Wageningen Food Safety Research, Wageningen University and Research, Wageningen 6708 WB, The Netherlands

*email corresponding author: laura.righetti@wur.nl

SI-1 – Stock solutions 2

SI-2 –Targeted analysis settings 3

SI-3 – MZmine settings...4

SI-4– R script 5

Table S 1: SIRIUS settings..7
Table S 2: Compound discoverer settings 7
Table S 3: Additional information of calibrants including the RT and linear range....10
Table S 4 False negatives NTS method - annotation with FSMS library 11

Figure S 1 Aligned, annotated and semi-quantified features for different extraction methods. A (ESI+) and B (ESI-) show the different features for the 6 extraction methods. C (ESI+) and D (ESI-) are a repeat of the extraction with QuEChERS and MeOH:ACN (N=8) to confirm that QuEChERS are the most favourable. Moreover, the low standard deviation indicates a good reproducibility of the extraction method. 12
Figure S 2 Extraction recovery (A) and matrix effect (B) for the calibrants. An extraction recovery above 50 (dashed line) is accepted. A matrix effect below 80% (red dashed line) indicates ion suppression and above 120% (red dashed line) ion enhancement. As can be observed , all compounds exhibited satisfactory recoveries (>55%), although most of them suffered from ion suppression. 13
Figure S 3 Evaluation of the different buffers (A) and organic solvents (B) tested during the development of the NTS HPLC-HRMS method based on the response

factors in logarithmic scale. From figure 3A it can be concluded that the blue triangle corresponding to ammonium acetate in MeOH results in the highest response factor for 16 out of 24 compounds. When testing ammonium acetate in different organic modifiers (3B) the conclusion was drawn that ammonium acetate in MeOH, 0.1% FA (3B, red circle) still results in the highest response factor and was therefore chosen as the mobile phase. 13
Figure S 4 comparison of the ion trap and orbitrap to acquire MS2. A) features aligned with ion trap MS2 (blue) and orbitrap (orange). Showing that more features were aligned when acquiring MS2 with the ion trap. B) percentage of features annotated in comparison to all the aligned features. Showing a higher percentage of annotation when using the orbitrap for MS2 than when using the ion trap for MS2. .14
Figure S 5 Extracted ion chromatogram of matrine acquired with the Orbitrap IQ-X Tribrid instrument. The figure shows the overlay of the EIC (m/z 249.1963, [M+H]+) of matrine in one bee pollen sample (BP67, black), in the analytical standard (red) and in the bee pollen samples spiked with the standard (blue). The chromatogram shows clearly two peaks in the spiked bee pollen sample (blue). The first peak matches with the analytical standard (red) whereas the second peak matches with the sample (black). Indicating that the compound in the sample is not matrine but most likely an isomer of matrine (e.g., leontine). 15
Figure S 6 this figure shows the matching MS2 spectra of the EIC presented in figure S5. In the first row the MS2 of the bee pollen sample 67. It can be observed that in the MS2 of BP67 an additional peak is present when comparing to the analytical standard (2nd row), supporting the hypothesis that the compound in BP67 is not matrine. Moreover, when looking at the spiked bee pollen (3rd row), multiple MS2 spectra were obtained (highlighted with * and **). Although, the standard has been spiked onto this sample, it is clear that fragments of m/z 114 and 136 present in both acquired MS2 making it difficult to distinguish matrine and its isomer solely based on its MS2. 16
Figure S 7 Extracted Ion Chromatogram of fenpropidin acquired with the Orbitrap IQ-X Tribrid instrument. The figure shows the overlay of the EIC (m/z 274.2519, [M+H]+) of fenpropidin in one bee pollen sample (BP67, red), in the analytical standard (blue) and in the bee pollen samples spiked with the standard (black). The chromatogram shows clearly two peaks in the spiked bee pollen sample (black) with good baseline separation. The first peak matches with the analytical standard (blue) whereas the second peak matches with the sample (red). Strongly Indicating that the compound in the sample is not fenpropidin but most likely an isomer. 17
Figure S 8 A) Comparison between predicted and expected concentrations of Validators. The comparison reveals a trend in which most compounds are being underpredict when using the prediction model MS2quant B) Set concentration range when using the average fold error of 2.5.This figure highlights that using the average fold error to set the concentration range is not cautious enough as the expected concentration lays outside the range for 3 out of 8 compounds. 18

SI-1 – Stock solutions

A stock solution (Stock 1) of 10 mg/L for each compound was prepared to develop, optimize and validate the NTS HPLC-HRMS method. Stock 1 contained the following compounds: senecionine, lasiocarpinie, and trachelanthamine purchased from Phytoplan; deoxynivalenol (DON), zearalenone (ZEN), aflatoxin B1 purchased from Romer Labs; fumonisin B1, boscalid, carbendazim, imidacloprid, coumaphos, PFOA, linoleic acid, m-coumaric acid, myricetin, L-phenylalanine, rosmarinic acid, Rutin, secoisolariciresinol, and chlorpyrifos purchased from Sigma-Aldrich; Thiacloprid, and Carbaryl purchased from HPC.

A second stock solution (Stock 2) was prepared to spike the validation sample (VAL). Containing the following analytical standards: spartiodine N-oxide, heliotrine, and integerrimine (Phytoplan); acetamiprid, and phosmet (Sigma-Aldrich); diphenyleamine (HPC); ochratoxin A (OTA), and T2-toxin (Romer labs). The concentrations of the compounds in stock 2 are according the table below

Table 1 compound overview validators

Compound	Chemical formula	g/mol	LogP	Conc. in stock 2 (µg/L)	Conc. in BP extract (VAL) (µg/L)
T-Toxin	$C_{24}H_{34}O_9$	466.5	0.90	1500.6	33.35
Aflatoxin M1	$C_{17}H_{12}O_7$	328.27	0.50	225.1	5.00
Ochratoxin A	$C_{20}H_{18}ClNO_6$	403.80	4.70	1501.8	33.37
Spartioidine N-oxide	$C_{18}H_{23}NO_6$	349.40	0.10	1500.6	33.35
Heliotrine	$C_{16}H_{27}NO_5$	313.99	0.40	1500.6	33.35
Integerrimine	$C_{18}H_{25}NO_5$	335.40	1.10	1500.6	33.35
Acetamiprid	$C_{10}H_{11}ClN_4$	222.68	0.80	15396.2	342.14
Diphenylamine	$C_{12}H_{11}N$	162.22	3.50	1500.6	33.35
Phosmet	$C_{11}H_{12}NO_4PS_2$	317.30	2.95	150006.0	333.47
Captan	$C_9H_8Cl_3NO_2S$	300.60	2.80	1500.6	33.35

SI-2 –Targeted analysis settings

Pesticides: The targeted analysis for 326 pesticides was done in MRM mode on a Sciex TQ6500+ system in positive ionization mode. The collision energy ranged between 4 – 73 eV depending on the pesticide. The column used was a BEH-C18 (1.7µm), 100 x 2.1 mm (Waters). The mobile phase consisted of 5 mM ammonium formate in MiliQ was used (solvent A) and 100% MeOH (solvent B). The total run time was 15 minutes starting at 10% B and increased to 90% B in a span of 12 minutes. It was hold on 90% B for 1 minute before returning back to 10% B where it equilibrated for 2 minutes before the next injection. The flow rate was set to 0.45 mL/min with a column temperature of 40 ºC. The sample tray was set to 10 ºC and the injection volume was set to 1 µL.

The samples were extracted with the described QuEChERS method.

Pyrrolizidine alkaloids: The targeted analysis of 78 pyrrolizidine alkaloids (PA) was done in MRM mode (ESI+) on a Xevo TQ-XS system connected to a Acquity UPLC I-class plus. The collision energy ranged between 25-40 eV depending on the PA. The column used was a Raptor Inert ARC-C18 (2.7 µm), 100 x 2.1 mm connected to a matching 5 mm guard column (RESTEK). As mobile phase HPLC-MS grade water was used with 0.1% FA (solvent A) and acetonitrile, 0.1% FA (solvent B). The total run time was 14 minutes starting at 4% B which was hold for 5.5. After 5.5 minutes the percentage B slowly increased in 5.5 min to 25% B. From there the percentage B was increased to 95% in 0.5 min. After a hold of 1 min at 95% B the gradient was returned to starting conditions in 0.5 min to equilibrate for 1min before the next injection. The flow rate was 0.4 mL/min and the column temperature was set to 25 ºC. The sample tray was set to 10 ºC and the injection volume was set to 1 µL

Samples were extracted as followed: After adding 10mL of MiliQ, 0.2% FA to 0.5 gram bee pollen the samples were shaken at an overhead shaker for 30 min at 300 rpm followed by 15 minutes of centrifugation at 3500 rpm. The samples were cleaned using a C18 SPE cartridge. The eluent was dried under a stream of nitrogen while in water bath of 50 ºC. The dried extracts were then reconstituted in 500 µL MeOH. Prior to analysis the extracts were added to a 0.45 µm filter vial.

SI-3 – MZmine settings

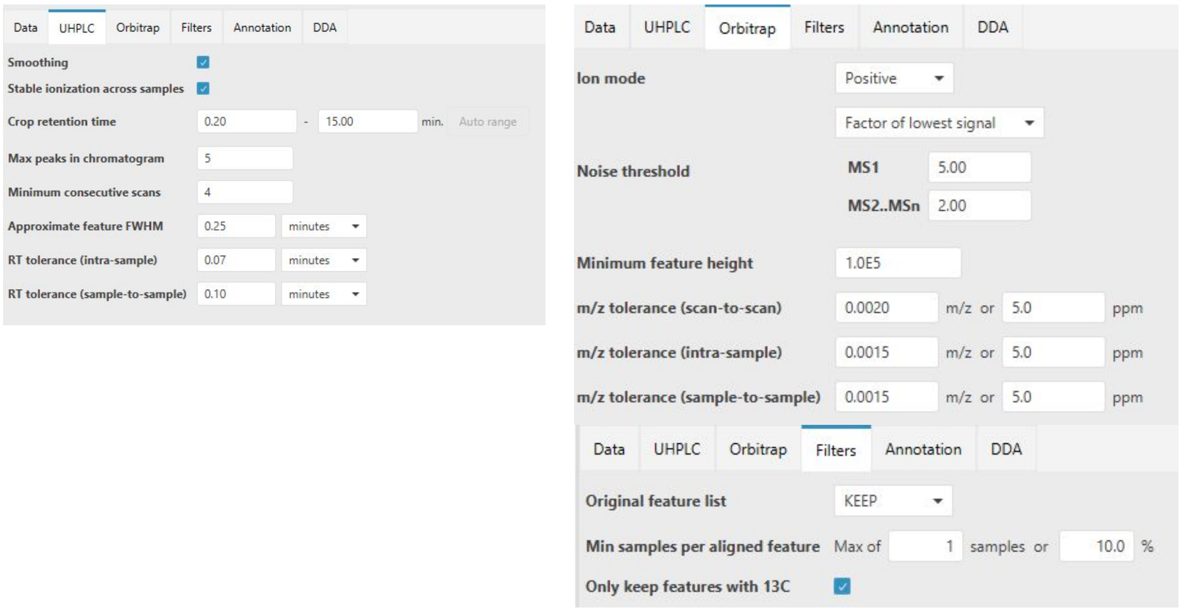

**the settings for negative ion mode are identical*

SI-4– R script

Filtering SIRIUS output and aligning MS2quant input

S.Heeren

2025-06-06

*this script was written with the help of ChatGPT

library(readxl); library(dplyr); library(writexl); library(openxlsx)

Aligning of different scores

#files needed: #formula_identifications.xlsx > tree score and medain absolute max error #compound_identifications.xlsx > ConfidenceScore, CSI:FingerIDScore, ZodiacScore, SiriusScore, smiles, name #canopus_formula_summary.xlsx > the featureId's, "NPC#pathway", "NPC#superclass", "NPC#class"

from tsv to excel

```
data <- read.delim("formula_identifications.tsv")
write_xlsx(data, "formula_identifications.xlsx")

data <- read.delim("compound_identifications.tsv")
write_xlsx(data, "compound_identifications.xlsx")

data <- read.delim("canopus_formula_summary.tsv")
 write_xlsx(data, "canopus_formula_summary.xlsx")
```

Aligning SIRIUS data

```
data <- read_excel("canopus_formula_summary.xlsx", sheet = "Sheet1")
column_to_copy <- data$featureId
new_data <- data.frame(ColumnName = column_to_copy)
colnames(new_data) <- "featureId"
write_xlsx(new_data, "merged_file.xlsx")

sheet1 <- read_excel("merged_file.xlsx", sheet = "Sheet1")
sheet2 <- read_excel("formula_identifications.xlsx", sheet = "Sheet1")

sheet2_selected <- sheet2 %>% select(featureId, TreeScore,
"medianAbsoluteMassErrorFragmentPeaks.ppm.")
merged_data <- left_join(sheet1, sheet2_selected, by = "featureId")
write_xlsx(merged_data, "merged_file.xlsx")

sheet1 <- read_excel("merged_file.xlsx", sheet = "Sheet1")
sheet3 <- read_excel("compound_identifications.xlsx", sheet = "Sheet1")

sheet3_selected <- sheet3 %>% select(featureId, ConfidenceScore, "CSI.FingerIDScore",
ZodiacScore, SiriusScore,
            smiles, name)
merged_data <- left_join(sheet1, sheet3_selected, by = "featureId")
write.xlsx(merged_data, "merged_file.xlsx", overwrite = TRUE)
```

```
sheet1 <- read_excel(“merged_file.xlsx”, sheet = “Sheet 1”)
sheet4 <- read_excel(“canopus_formula_summary.xlsx”, sheet = “Sheet1”)

sheet4_selected <- sheet4 %>% select(featureId, “NPC.pathway”, “NPC.superclass”, “NPC.class”,
        “molecularFormula”, “adduct”, “precursorFormula”)
merged_data <- left_join(sheet1, sheet4_selected, by = “featureId”)
write.xlsx(merged_data, “merged_file.xlsx”, overwrite = TRUE)
```

CLEANING OF SIRIUS output (MERGED_FILE)

```
data <- read.xlsx(“merged_file.xlsx”, sheet = 1)
data$"medianAbsoluteMassErrorFragmentPeaks.ppm." <-
        as.numeric(data$“medianAbsoluteMassErrorFragmentPeaks.ppm.”)
data$"ConfidenceScore" <- as.numeric(data$“ConfidenceScore”)

filtered_data <- data[!is.na(data$“ConfidenceScore”), ]
write.xlsx(filtered_data, “merged_file_cleaned.xlsx”, overwrite = TRUE)

data <- read.xlsx(“merged_file_cleaned.xlsx”, sheet = 1)
filtered_data <- data[data$“CSI.FingerIDScore” >= -150, ]
write.xlsx(filtered_data, “merged_file_cleaned.xlsx”, overwrite = TRUE)

data <- read.xlsx(“merged_file_cleaned.xlsx”, sheet = 1)
filtered_data <- data[data$“medianAbsoluteMassErrorFragmentPeaks.ppm.” <= 5, ]
write.xlsx(filtered_data, “merged_file_cleaned.xlsx”, overwrite = TRUE)
```

alignment MS2Quant intput

```
#peak area and retention times
#BEFORE YOU START RUNNING THIS PART MAKE SURE THAT THE XXX_POS….XLSX HAS
THE RIGHT COLUMN HEADINGS.
 data <- read_excel(“merged_file_cleaned.xlsx”, sheet = “Sheet 1”)
columns_to_copy <- data[, c(“featureId”, “smiles”)]
write.xlsx(columns_to_copy, “MS2Quant_input.xlsx”)

data <- read_excel(“MS2Quant_input.xlsx”)
colnames(data)[colnames(data) == “featureId”] <- “row ID”
print(data)
write.xlsx(data, “MS2Quant_input.xlsx”, overwrite = TRUE)

sheet1 <- read_excel(“MS2Quant_input.xlsx”, sheet = “Sheet 1”)
sheet2 <- read_excel(“XXX_POS_iimn_gnps_quant.xlsx”, sheet = “Sheet1”) #row retention time,
Peak_area sheet1_selected <- sheet1 %>% select(‘row ID’)
sheet2_selected <- sheet2 %>% select(“row ID”, “row m/z”, “row retention time”, “put samples here”,)

merged_data <- left_join(sheet1, sheet2_selected, by = “row ID”)
write.xlsx(merged_data, “MS2quant_input.xlsx”, overwrite = TRUE)
```

***Table S 1**: SIRIUS settings*

Parameter	Setting
Instrument	Orbitrap
Filter by isotope pattern	yes
MS2 mass accuracy (ppm)	5
MS/MS isotope scorer	IGNORE
Candidates scored	10
Min canidadates per ion stored	1
Use DB formulas only	CHEBI; COCONUT; GNPS; HMDB; KEGG; KNApSAcK; Maconda; NORMAN; Natural Products; Plantcyc
Possible Ionizations	All
Tree timeout	0
Compound timout	0
Use heuristic aboce m/z	3000
Use heuristic only above m/z	650
SIRIUS - Elements allowed in molecular formula	Default settings
ZODIAC	Default settings
Predict FPs – adducts	NEG: [M+Br]- ; [M+Cl]- ; [M-H]- ;<br>[M+CH2O2 – H]- ;<br>[M+C2H3N-H]-<br>POS: [M+H]+; [M+K]+; [M+Na]+;<br>[M+H3N+H]+;
CSI:FingerID – Structure database search	Default settings
CANOPUS	selected

***Table S 2:** Compound discoverer settings*

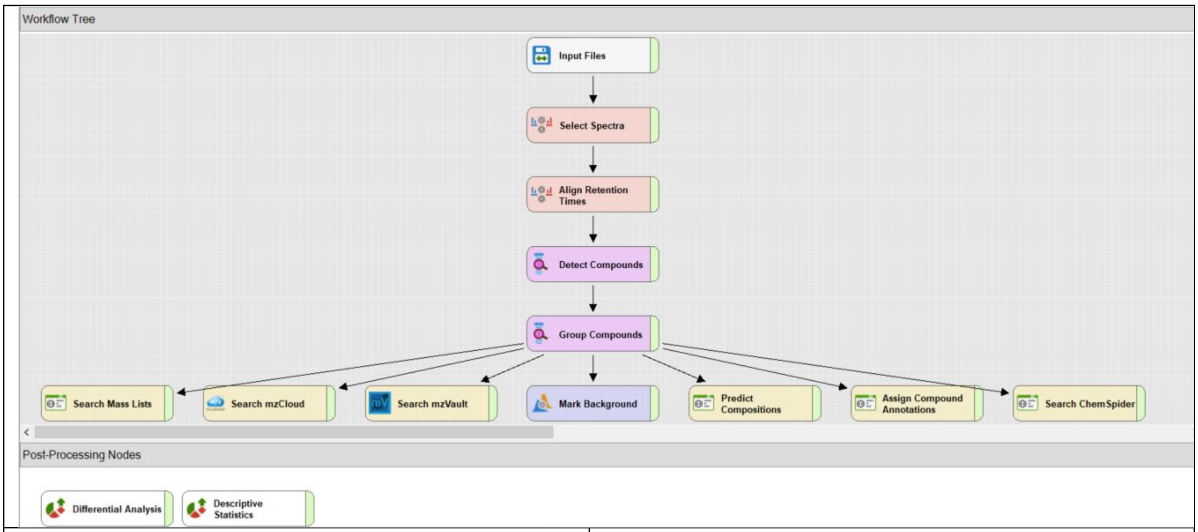

**Select Spectra**	**Mark Background**
Lower RT Limit: 0<br>Upper RT Limit: 0<br>Polarity mode: Any	Max. Sample/blank: 5<br>Max. Blank/Sample: 0<br>Hide Background: true

Detect compounds
Mass Tolerance: 3 ppm
Min. Peak Intensity: 10000
Use Most Intense: True
Precursor Mass: 0.025 Da
Chromatographic: 1.5
Remove Baseline: False
Group Isotopes: Br, Cl
Ions: all

Group Compounds
Mass tolerance: 3 ppm
RT Tolerance [Min]: 0.2
Minimum Valley [%]: 10
Align Peaks: false
Preferred ions: [M+H]+; [M+NH4]+; [M-H]-
Area contribution: 3
CV Contribution: 10
FWHM to Base Contribution: 5
Jaggedness Contribution: 5
Modality Contribution: 5
Zig-Zag Index Contribution: 5
Peak Rating Threshold: 3
Number of Files: 3

Search mzCloud
Compound classes: all
Library: Autoprocessed; Reference
Search MSn Tree: true
DDA search:
Identity search: Cosine
Match Activation Type: true
Match Activation Energy: Match with Tolerance
Activation Energy Tolerance: 20
Similarity Search: Confidence Forward
Match Factor Threshold:50
DIA:
Use DIA Scans for Search: False
Max/ Isolation Width [Da]: 500
Match Activation Type: False
Match Activation Energy: Any
Activation Energy Tolerance: 100
Apply Intensity Threshold: False
Match Factor Threshold: 20

Search Chemspider
Databases: BioCyc; FDA; FooDB: Human Metabolome Database; KEGG; MassBank
Search mode: By Formula or Mass
Mass Tolerance: 5 ppm
Max # of results per compound: 100
Max # of predicted compositions to be searched per compound: 5

Search Mass Lists
Mass lists: HRMS database dbm
Use Retention Time: true
RT Tolerance: 2min
Mass Tolerance 5 ppm

Search mzVault
mzVault Library: FoodSafetyMassSpectalLibrary_V2_25.db
compound classes: all
match ion activation type: true
match ion activation energy: match with tolerance
ion activation energy tolerance: 20
Match ionization method: true
Apply intensity threshold: true
Precursor mass tolerance: 10 ppm
Match analyser type: true
Search algorithm: HighChem HighRes
Match factor threshold: 50
RT tolerance [min] 2
Use Retention time: False

Predicted Compositions
Mass Tolerance: 3ppm
Min Element Counts: CH
Max Element Counts: C90 H190 Br3 Cl5 F25 N10 O18 P5 S5
Min RDBE: 0
Max RDBE: 40
Min. H/C: 0.1
Max. H/C: 3.5
Max # candidates: 10
Intensity tolerance: [5]: 30
Intensity threshold [%]: 0.1
S/N threshold: 3
Use Dynamic Recalibration: true
Use fragment matching: true
Mass tolerance: 5ppm
S/N threshold: 3

Assign Compound Annotations
Mass Tolerance: 5 ppm
Data Source #1: mzVault
Data Source #2: mzCloud
Data Source #3: MassList Search
Data Source #4: ChemSpider Search
Data Source #5: Predicted Compositions
Use mzLogic: true
Use Spectral distance: true
Shift threshold: 20
Shift range: 20
Clear names: false

Differential Analysis
Log10 Transform Values: true
Update Peak Rating: True
Area contribution: 3
CV contribution: 10
FWHM to Base contribution: 5
Jaggedness contribution: 5
Modality contribution: 5

Zig-Zag Index contribution: 5

Table S 3: *Additional information of calibrants including the RT and linear range*

Calibrant	Molecular Formula	LogP	Molecular ion	Adduct	RT (min)	Linear range (µg/L)	
						Lower limit	Upper limit
Phenyl alanine	$C_9H_{11}NO_2$	-1.38	166.0863	[M+H]+	4.50	5	1000
Senecionine	$C_{18}H_{25}NO_5$	-0.88	336.1805	[M+H]+	6.95	2.5	100
Deoxynivalenol	$C_{15}H_{20}O_6$	-0.70	297.1333	[M+H]+	6.01	10	100
Fumonisin B1	$C_{34}H_{59}NO_{15}$	-0.50	722.3957	[M+H]+	9.92	10	100
			720.3812	[M-H]-	10.01	50	1000
Lasiocarpine	$C_9H_{10}ClN_5O_2$	0.50	412.2330	[M+H]+	8.17	0.25	100
Imidacloprid	$C_9H_{10}ClN_5O_2$	0.57	256.0596	[M+H]+	7.07	1.25	250
Thiacloprid	$C_{10}H_9ClN_4S$	1.26	253.0309	[M+H]+	7.99	1.25	500
Trachelanthamine	$C_{15}H_{27}NO_4$	1.40	286.2008	[M+H]+	6.03	1.25	500
Carbendazim	$C_9H_9N_3O_2$	1.52	192.0770	[M+H]+	6.60	1.25	500
Aflatoxin B1	$C_{17}H_12O_6$	1.60	313.0707	[M+H]+	9.05	0.25	100
Carbaryl	$C_{12}H_{11}NO_2$	2.36	202.0862	[M+H]+	9.37	2.5	500
Boscalid	$C_{18}H_{12}Cl_2N_2O$	2.96	343.0399	[M+H]+	10.62	1.25	50
Coumaphos	$C_{14}H_{16}ClO_5PS$	4.13	363.0212	[M+H]+	11.51	0.25	10
Chlorpyrifos	$C_9H_{11}Cl_3NO_3PS$	4.96	349.9336	[M+H]+	12.30	0.25	50
Zearalenone	$C_{18}H_{22}O_5$	2.90	317.1394	[M-H]-	10.97	1.25	500
PFOA	$C_8HF_{15}O_2$	4.90	412.9660	[M-H]-	10.67	1.25	500
m-Coumaric acid	$C_9H_8O_3$	1.80	163.0401	[M-H]-	7.90	1.25	500
Myricetin	$C_{15}H_{10}O_8$	1.20	317.0303	[M-H]-	8.51	5	2000
Rutin	$C_{27}H_{30}O_{16}$	1.30	609.1481	[M-H]-	8.06	2.5	1000
Rosmarinic acid	$C_{18}H_{16}O_8$	2.40	359.0789	[M-H]-	8.08	2.5	250
Secoisolariciresinol	$C_{20}H_{26}O_6$	2.5	361.1657	[M-H]-	12.00	-	-
Linoleic acid	$C_{18}H32O_2$	7.05	279.2330	[M-H]-	13.16	-	-
Soy LysoPC (18:2)	$C_{26}H_{50}NO_7P$	5	520.3392	[M+H]+	12.43	-	-
Soy LysoPC (16:0)	$C_{24}H_{50}NO_7P$	5.6	496.3399	[M+H]+	12.61	-	-

***Table S 4** False negatives NTS method - annotation with FSMS library*

Compound name	Molecular formula	Freestyle?	MS2 match?	SIRIUS?
Aflatoxin B2	$C_{17}H_{14}O_6$	yes	no	no
Azoxystrobin	$C_{22}H_{17}N_3O_5$	yes	yes	yes
Coniine	$C_8H_{17}N$	yes	no	no
Cyclohexanone oxime	$C_6H_{11}NO$	yes	yes	no
Diethyltoluamide; N,N-Diethyl-3-methylbenzamide; DEET	$C_{12}H_{17}NO$	no	no	no
Dimoxystrobin	$C_{19}H_{22}N_2O_3$	yes	no	no
Echiumine N-oxide	$C_{20}H_{31}NO_7$	yes	yes	Different PA
Glucobrassicanapin	$C_{12}H_{21}NO_9S_2$	yes	no	no
Gluconapin	$C_{11}H_{19}NO_9S_2$	yes	no	no
Gluconasturtiin	$C_{15}H_{21}NO_9S_2$	yes	no	no
Morphine	$C_{17}H_{19}NO_3$	yes	no	no
o-Toluidine	C_7H_9N	no	no	no
Papaverine	$C_{20}H_{21}NO_4$	yes	yes	Different isoquinoline alkaloid
Platyphylline	$C_{18}H_{27}NO_5$	yes	yes	Different PA
Pyraclostrobin	$C_{19}H_{18}ClN_3O_4$	yes	no	no
Semiamitraz; N'-(2,4-Dimethylphenyl)-N-methylformamidine	$C_{10}H_{14}N_2$	yes	inconclusive	no
Sparteine	$C_{15}H_{26}N_2$	yes	inconclusive	yes
Tebuconazole	$C_{16}H_{22}ClN_3O$	yes	no	no
Thebaine	$C_{19}H_{21}NO_3$	yes	yes	no
Thiamethoxam	$C_8H_{10}ClN_5O_3S$	yes	no	no
Trenbolone acetate	$C_{20}H_{24}O_3$	yes	yes	Different steroid
Trinexapac	$C_{11}H_{12}O_5$	yes	yes	Different chemical class
Dibutyl phthalate	$C_{16}H_{22}O_4$	yes	inconclusive	yes
Sophocarpine	$C_{15}H_{22}N_2O$	yes	yes	yes

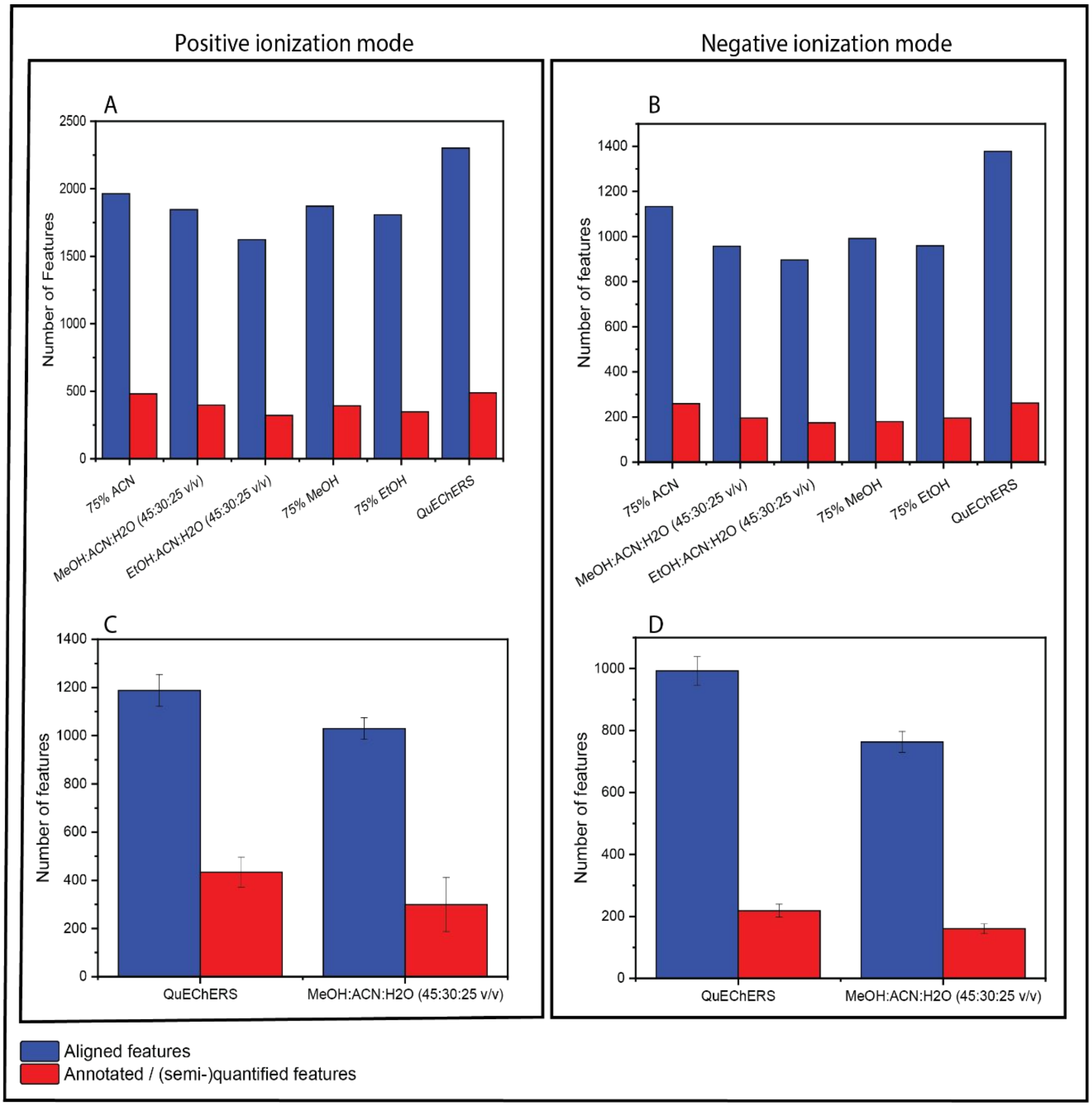

***Figure S 1** Aligned, annotated and semi-quantified features for different extraction methods. A (ESI+) and B (ESI-) show the different features for the 6 extraction methods. C (ESI+) and D (ESI-) are a repeat of the extraction with QuEChERS and MeOH:ACN (N=8) to confirm that QuEChERS are the most favourable. Moreover, the low standard deviation indicates a good reproducibility of the extraction method.*

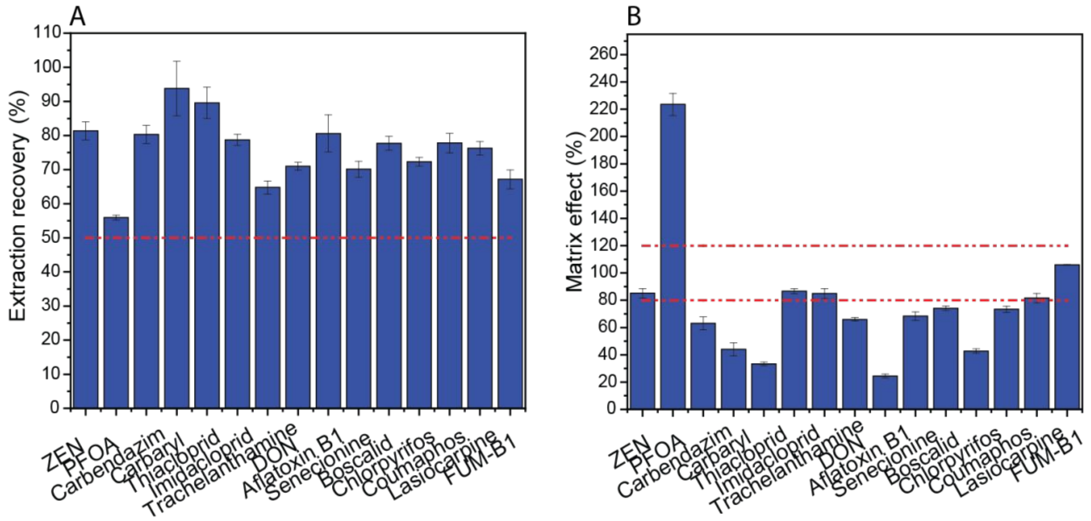

Figure S 2 *Extraction recovery (A) and matrix effect (B) for the calibrants. An extraction recovery above 50 (dashed line) is accepted. A matrix effect below 80% (red dashed line) indicates ion suppression and above 120% (red dashed line) ion enhancement. As can be observed , all compounds exhibited satisfactory recoveries (>55%), although most of them suffered from ion suppression.*

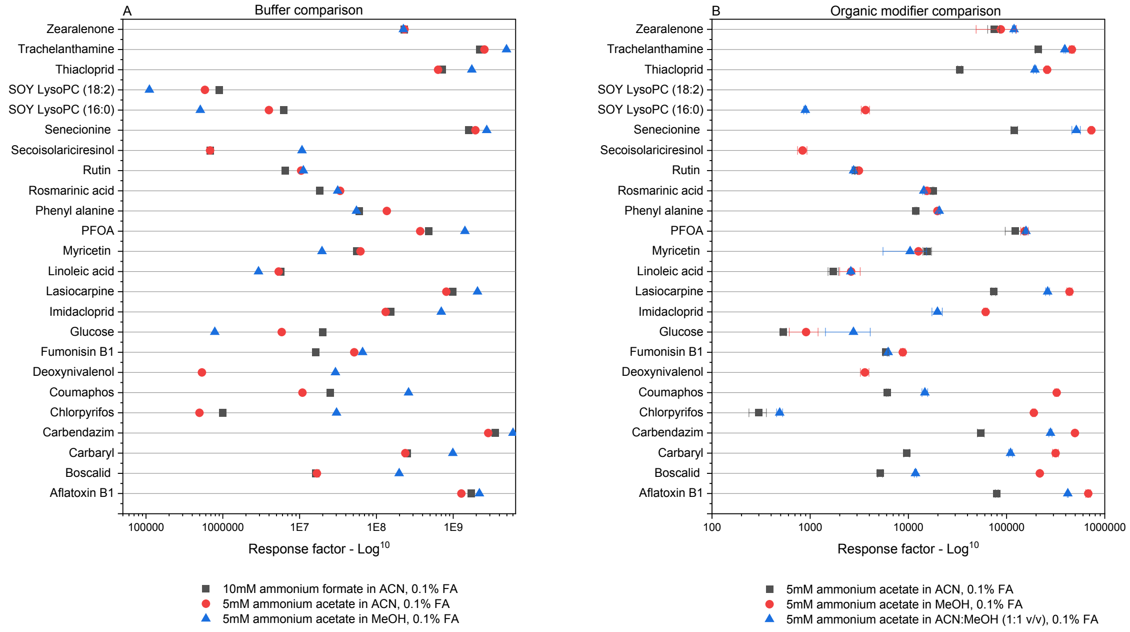

Figure S 3 *Evaluation of the different buffers (A) and organic solvents (B) tested during the development of the NTS HPLC-HRMS method based on the response factors in logarithmic scale. From figure 3A it can be concluded that the blue triangle corresponding to ammonium acetate in MeOH results in the highest response factor for 16 out of 24 compounds. When testing ammonium acetate in different organic modifiers (3B) the conclusion was drawn that ammonium acetate in MeOH, 0.1% FA (3B, red circle) still results in the highest response factor and was therefore chosen as the mobile phase.*

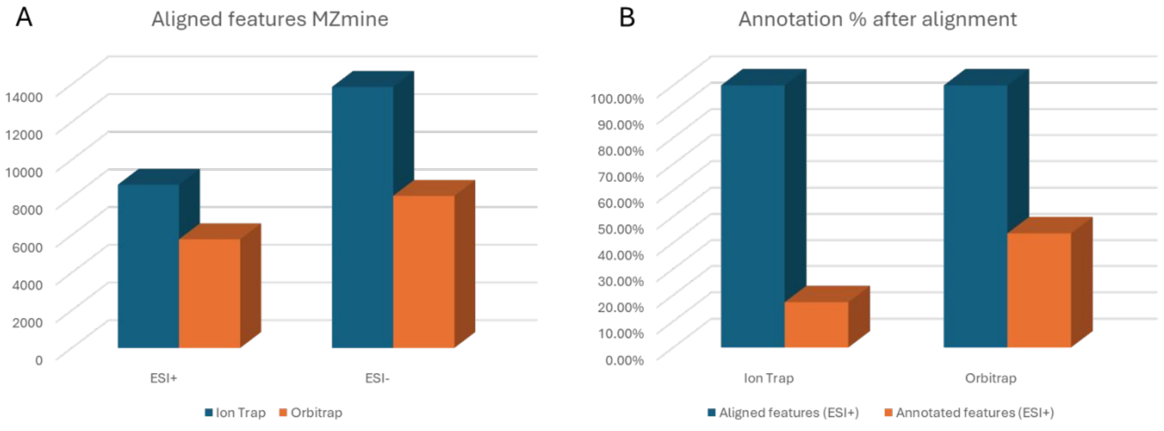

Figure S 4 *comparison of the ion trap and orbitrap to acquire MS2. A) features aligned with ion trap MS2 (blue) and orbitrap (orange). Showing that more features were aligned when acquiring MS2 with the ion trap. B) percentage of features annotated in comparison to all the aligned features. Showing a higher percentage of annotation when using the orbitrap for MS2 than when using the ion trap for MS2.*

.

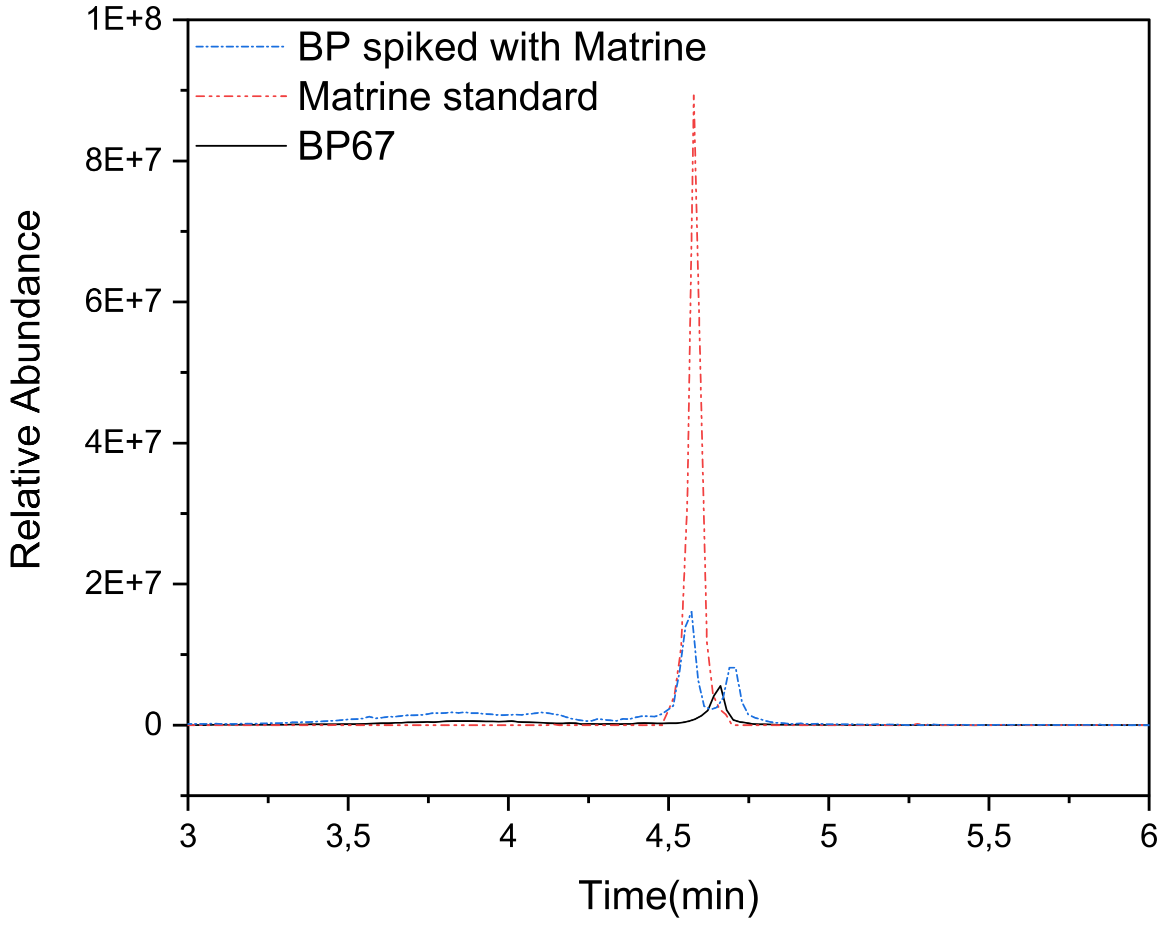

Figure S 5 *Extracted ion chromatogram of matrine acquired with the Orbitrap IQ-X Tribrid instrument. The figure shows the overlay of the EIC (m/z 249.1963, [M+H]+) of matrine in one bee pollen sample (BP67, black), in the analytical standard (red) and in the bee pollen samples spiked with the standard (blue). The chromatogram shows clearly two peaks in the spiked bee pollen sample (blue). The first peak matches with the analytical standard (red) whereas the second peak matches with the sample (black). Indicating that the compound in the sample is not matrine but most likely an isomer of matrine (e.g., leontine).*

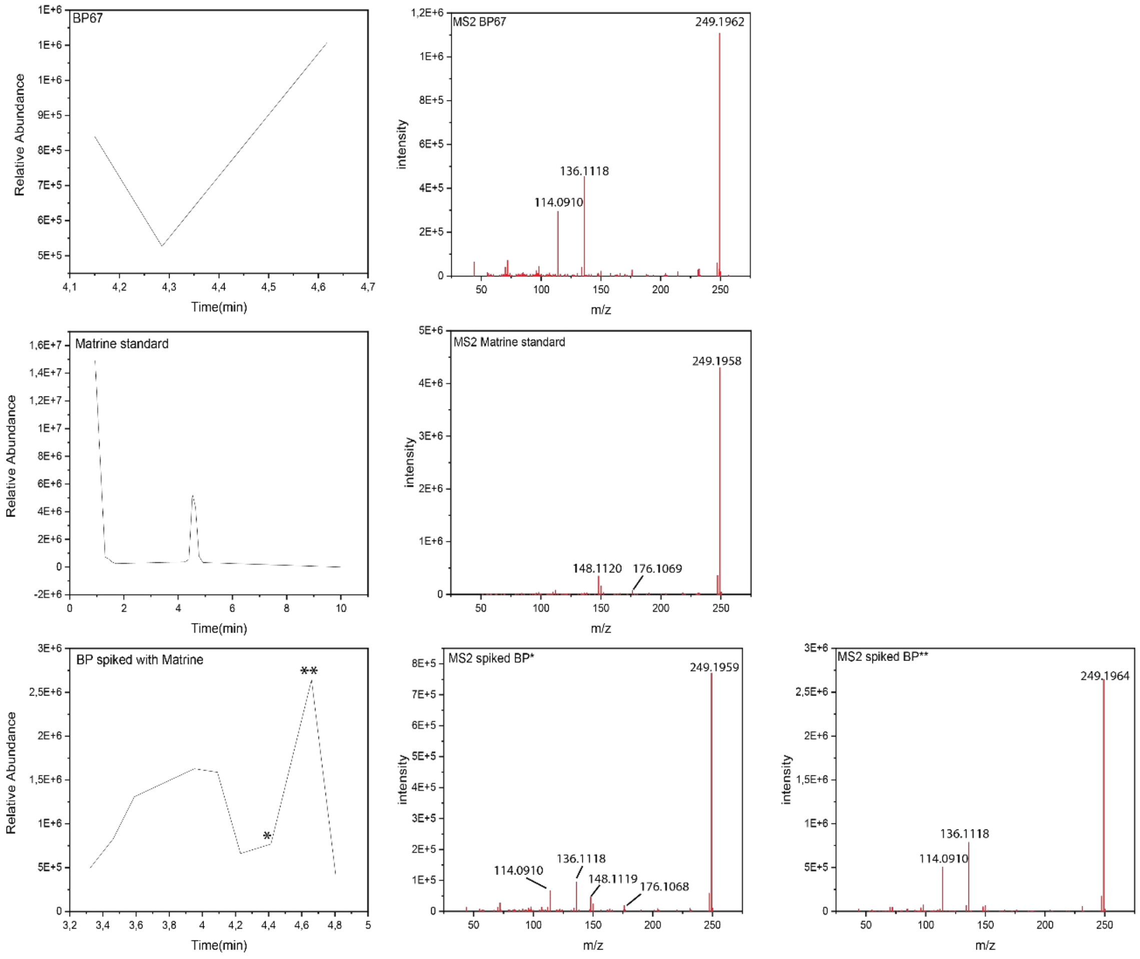

Figure S 6 *this figure shows the matching MS2 spectra of the EIC presented in figure S5. In the first row the MS2 of the bee pollen sample 67. It can be observed that in the MS2 of BP67 an additional peak is present when comparing to the analytical standard (2nd row), supporting the hypothesis that the compound in BP67 is not matrine. Moreover, when looking at the spiked bee pollen (3rd row), multiple MS2 spectra were obtained (highlighted with * and **). Although, the standard has been spiked onto this sample, it is clear that fragments of m/z 114 and 136 present in both acquired MS2 making it difficult to distinguish matrine and its isomer solely based on its MS2.*

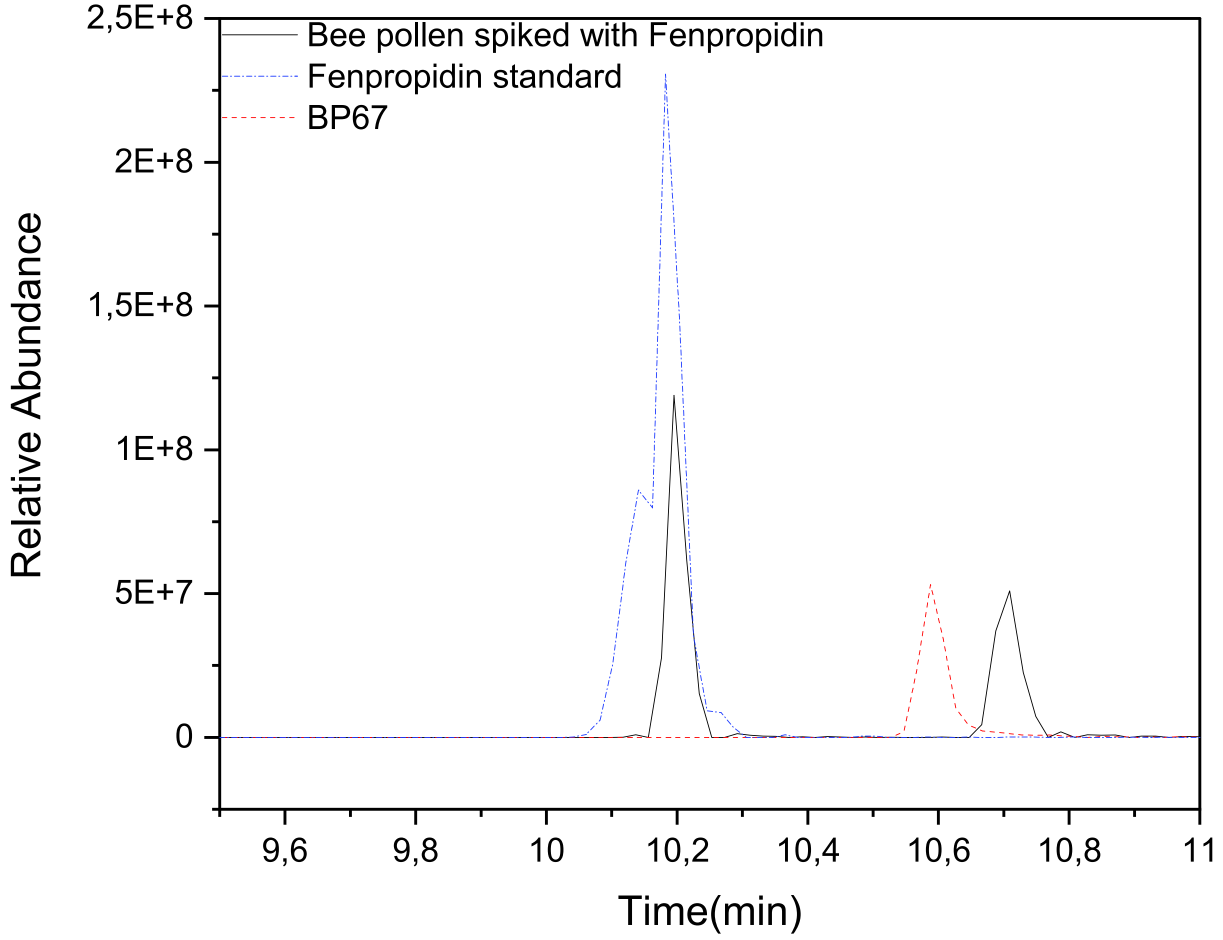

Figure S 7 *Extracted Ion Chromatogram of fenpropidin acquired with the Orbitrap IQ-X Tribrid instrument. The figure shows the overlay of the EIC (m/z 274.2519, [M+H]+) of fenpropidin in one bee pollen sample (BP67, red), in the analytical standard (blue) and in the bee pollen samples spiked with the standard (black). The chromatogram shows clearly two peaks in the spiked bee pollen sample (black) with good baseline separation. The first peak matches with the analytical standard (blue) whereas the second peak matches with the sample (red). Strongly Indicating that the compound in the sample is not fenpropidin but most likely an isomer.*

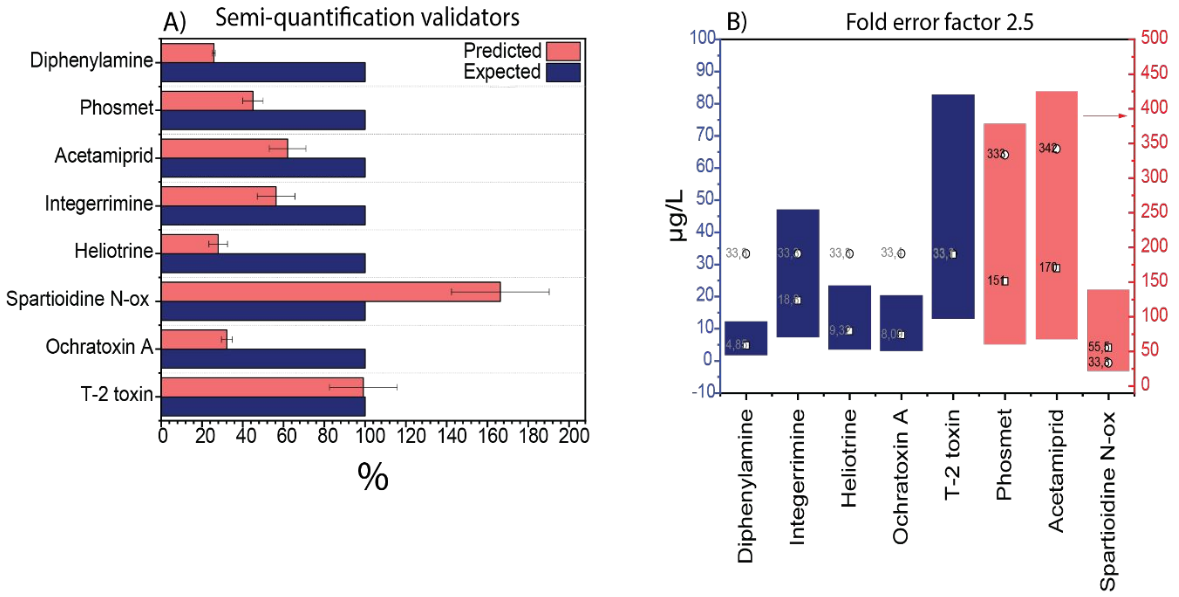

Figure S 8 *A) Comparison between predicted and expected concentrations of Validators. The comparison reveals a trend in which most compounds are being underpredict when using the prediction model MS2quant B) Set concentration range when using the average fold error of 2.5.This figure highlights that using the average fold error to set the concentration range is not cautious enough as the expected concentration lays outside the range for 3 out of 8 compounds.*
